# Multimodality Imaging of Intracardiac Lesions in a Lung Transplant Patient: Atypical Presentation of Culture-Negative Endocarditis

**DOI:** 10.1016/j.jaccas.2025.105862

**Published:** 2025-12-10

**Authors:** Ikeoluwapo Kendra Bolakale-Rufai, Cedrick Mutebi, Danish Saleh, Michael P. Angarone, Catherine Myers, Joseph Bailey, Mohamed Al-Kazaz

**Affiliations:** a McGaw Medical Center of Northwestern University, Chicago, Illinois, USA; b Division of Cardiology, Department of Medicine, School of Medicine and Public Health, University of Wisconsin-Madison, Madison, Wisconsin, USA; c Division of Infectious Diseases, Department of Medicine, Northwestern University Feinberg School of Medicine, Chicago, Illinois, USA; d Division of Pulmonology, Department of Medicine, Northwestern University School of Medicine, Chicago, Illinois, USA; e Division of Cardiology, Department of Medicine, Northwestern University Feinberg School of Medicine, Chicago, Illinois, USA; f Bluhm Cardiovascular Institute, Northwestern Memorial Hospital, Chicago, Illinois, USA

**Keywords:** aortic valve, cardiac thrombus, infective endocarditis, intracardiac lesion, vegetations

## Abstract

**Background:**

Intracardiac lesions in immunocompromised patients with negative blood culture pose a diagnostic challenge, particularly when both thrombus and infective endocarditis are possible etiologies.

**Case Summary:**

A young woman with a history of lung transplantation on immunosuppression and thrombophilia was found to have new hypermetabolic, mobile lesions involving the interventricular septum. Blood cultures were negative, and owing to prohibitive surgical risk, she was managed conservatively with antibiotics, with an excellent response.

**Discussion:**

This case highlights the diagnostic complexity and the role of multimodality imaging associated with defining the etiology and treatment plan for intracardiac lesions in a patient with multiple medical comorbidities.

**Take-Home Message:**

Culture-negative endocarditis requires a high index of suspicion in immunocompromised patients with thrombotic risk factors and inconclusive microbiological and echocardiographic findings.

## Visual Summary

**Figure:**
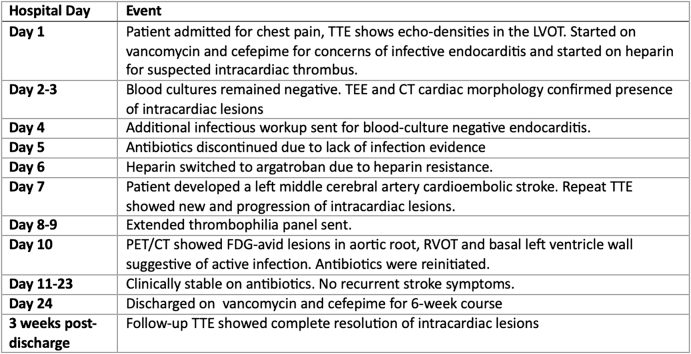

**Visual Summary:**
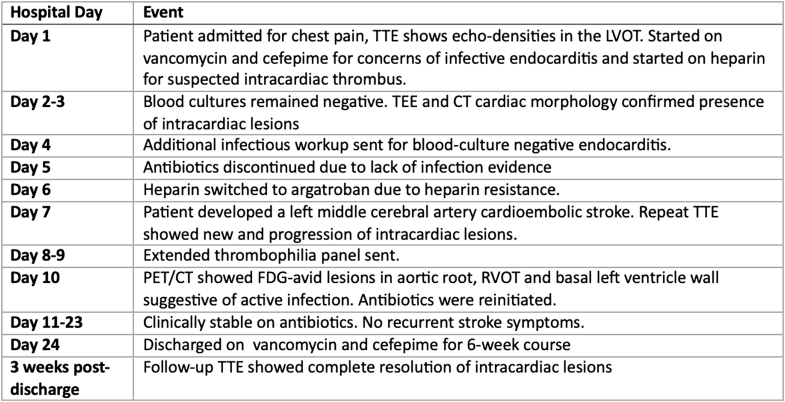
Timeline of Case CT = Computed tomography; FDG = Fluorodeoxyglucose; LVOT = Left ventricular outflow tract; PET = Positron emission tomography; RVOT = Right ventricular outflow tract; TEE = Tranesophageal echocardiography; TTE = Transthoracic echocardiography.

## History of Presentation

**Figure 1:**
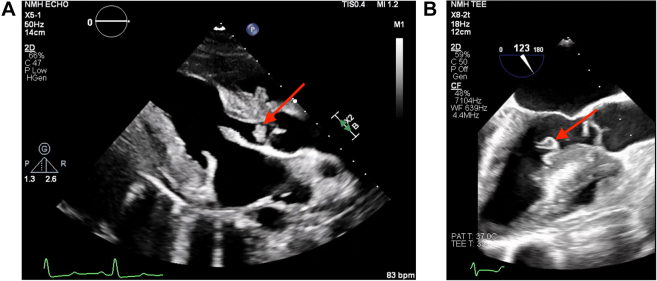
Echocardiographic Visualization of Intracardiac Lesions (A) Transthoracic echocardiography showing round mobile echodensity attached to the aortic valve (arrow). (B) Transesophageal echocardiography showing large echodensity attached to the basal anteroseptal region of the left ventricle (arrow).

A 34-year-old woman with a history of factor V Leiden mutation and bilateral orthotopic lung transplantation presented to the emergency department with substernal, nonexertional chest pain. She was hemodynamically stable on presentation with initial transthoracic echocardiography (TTE) revealing a 10 mm × 6 mm round, mobile echodensity on the left ventricular outflow tract (LVOT) with possible extension into the right ventricular outflow tract (RVOT) (Figure 1A).

**Take-Home Messages**

• Culture-negative endocarditis requires a high index of suspicion in immunocompromised patients with thrombotic risk factors and inconclusive microbiological and echocardiographic findings.
• Echocardiographic is highly sensitive for IE, but features of IE may overlap with intracardiac thrombus, NBTE, and cardiac tumors; therefore, multimodal imaging such as cardiac magnetic resonance and PET/CT can guide treatment decisions in these uncertain cases.

## Past Medical History

The patient's past medical history included bronchopulmonary dysplasia and acute respiratory distress syndrome secondary to meconium aspiration in infancy, complicated by recurrent pneumonia and end-stage emphysema, ultimately requiring a bilateral orthotopic lung transplantation, which was performed 17 months before presentation. Her post-transplant course included recurrent right bronchial stenosis that required endobronchial stenting and a complicated prolonged hospitalization. Her immunosuppressive medications included tacrolimus, mycophenolate, and prednisone. Also notable was her history of recurrent nonprovoked deep vein thromboses, including a bilateral iliac vein deep vein thrombosis for which she had a thrombectomy in the setting of a diagnosed factor V Leiden thrombophilia. She was on long-term anticoagulation with apixaban with good adherence.

## Differential Diagnosis

The differential diagnosis included a primary or secondary cardiac tumor (especially given use of immunosuppression), infective endocarditis (IE), nonbacterial thrombotic endocarditis (NBTE), and intracardiac thrombus. Given the presenting symptom of chest pain, other differential diagnoses included acute coronary syndrome, pneumonia, and pulmonary embolism.

## Investigations

Laboratory values on hospital admission were notable for leukocytosis 13.3 × 10^9^/L, elevated C-reactive protein 116 mg/L, high-sensitivity troponin 323 pg/mL (448 pg/mL peak), and B-type natriuretic peptide 178 pg/mL. Metabolic panel was normal, respiratory pathogen panel was negative, and sequential blood cultures remained sterile throughout hospitalization. Electrocardiography showed normal sinus rhythm with new T-wave inversions in leads II, III, and aVF. Chest x-ray was unremarkable for an acute cardiopulmonary process. Computed tomography (CT) angiography showed no evidence of acute pulmonary embolism, and the stent within the right bronchus intermedius was noted to be stable.

## Management

Owing to concerns for infection, empirical vancomycin and cefepime were started before blood culture collection. Concurrently, heparin was started in consideration of an intracardiac thrombus (pending further imaging at that time point), which was subsequently transitioned to argatroban because of heparin resistance. A cardiothoracic surgeon evaluated the patient; however, no surgical intervention was planned due to her clinical stability, negative cultures, and high-risk surgical candidacy. An infectious disease physician recommended discontinuing antibiotics on day 5 given the unremarkable work-up, and anticoagulation was continued. Two days after stopping antibiotics, the patient experienced a left middle cerebral artery stroke thought to be cardioembolic in origin. Repeat blood cultures were negative, and repeat TTE showed a new RVOT mobile echodensity measuring 10 mm × 10 mm.

**Figure 2:**
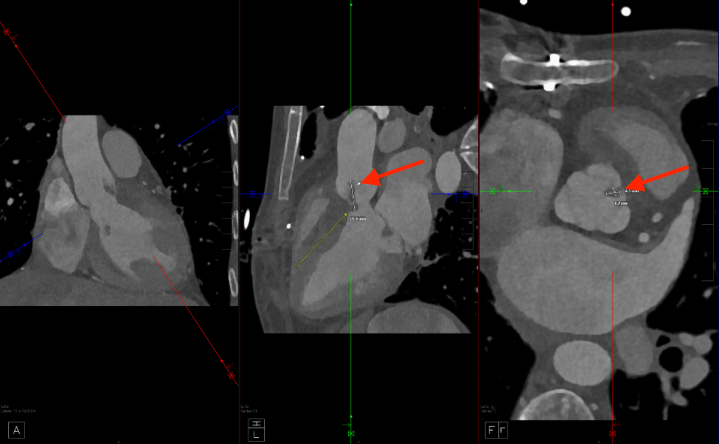
Aortic Valve Lesion Extending Into Left Ventricular Outflow Tract on Cardiac Computed Tomography Cardiac computed tomography showing filling defect attached to right aortic valve leaflet extending into the left ventricular outflow tract (arrow).

To further characterize the cardiac lesions, the patient underwent transesophageal echocardiography (TEE), which demonstrated an ejection fraction of 60% with large mobile echodensity attached to the basal anteroseptum of the left ventricle and a small 6 mm × 4 mm mobile echodensity attached to the aortic valve between the right and left coronary cusps (Figure 1B). The basal anteroseptum was also noted to be thickened and to involve the right ventricle. These findings were not present on her intraoperative TEE 17 months ago. CT cardiac morphology revealed a 5 × 3 × 20 mm filling defect attached to the right aortic valve leaflet extending into the LVOT and another 6 × 3 × 9 mm mobile filling defect on the basal anteroseptum of the left ventricle extending to the proximal LVOT during systole (Figure 2). Given the concern for culture-negative endocarditis, a broadened infectious disease work-up was obtained including tests for *Coxiella, Brucella, Bartonella,* histoplasmosis, and blastomycosis along with a Karius Test, all of which yielded negative results.

Given her history of thrombosis, absence of infectious symptoms, and negative infectious disease work-up, the hematologist was concerned that the lesion was a thrombus. Although there was no clear precipitating factor for thrombus formation, and her known factor V Leiden mutation alone would not explain the extent of thrombosis observed, there was a possibility of an underlying primary or secondary hypercoagulable state. As a result, an extended thrombophilia panel was performed, including testing for beta-2-glycoprotein, antiphospholipid syndrome antibody, lupus anticoagulant, anticardiolipin antibodies, prothrombin G20210A gene mutation, and antithrombin III, all of which were negative.

**Figure 3:**
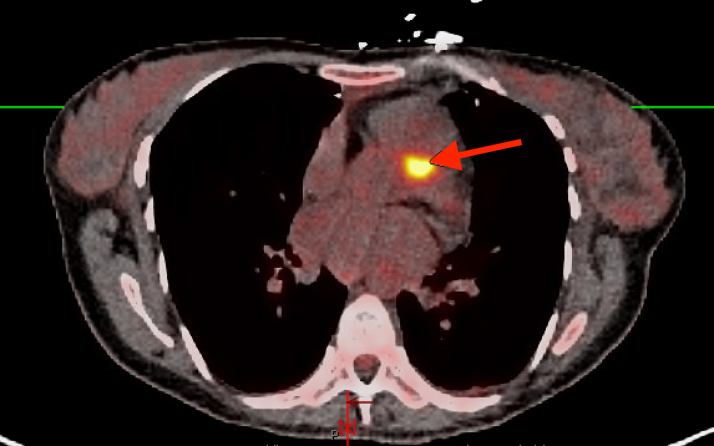
Positron Emission Tomography/Computed Tomography Visualization of Hypermetabolic Intracardiac Lesion Positron emission tomography/computed tomography showing area of increased metabolic activity along anterior aortic root (arrow).

Multimodality imaging plays an important role in management decisions and prognostication. Given concerns by primary teams and hematology, positron emission tomography (PET)/CT with fluorodeoxyglucose (FDG) was obtained for further evaluation and showed contiguous inflammatory metabolic activity along the anterior aortic root extending into the RVOT, consistent with multifocal infected vegetations and aortic root inflammation (Figure 3). An additional focus of increased FDG activity was noted along the basal lateral left ventricular myocardium adjacent to the mitral annulus, suggesting an additional nidus of infection. PET/CT effectively ruled out thrombus given the metabolic activity noted. Furthermore, the timeline of the presentation made culture-negative IE rather than malignancy the most likely diagnosis.

Cardiac surgery was deferred again owing to high operative risk. Percutaneous biopsy was considered because of potential for malignancy but was deferred owing to high risk of proximate friable tissue and low yield. After TEE, vancomycin and cefepime were reinitiated, and she was discharged home on these antibiotics via outpatient antimicrobial therapy coordinated with home health services.

## Outcomes and Follow-Up

**Figure 4:**
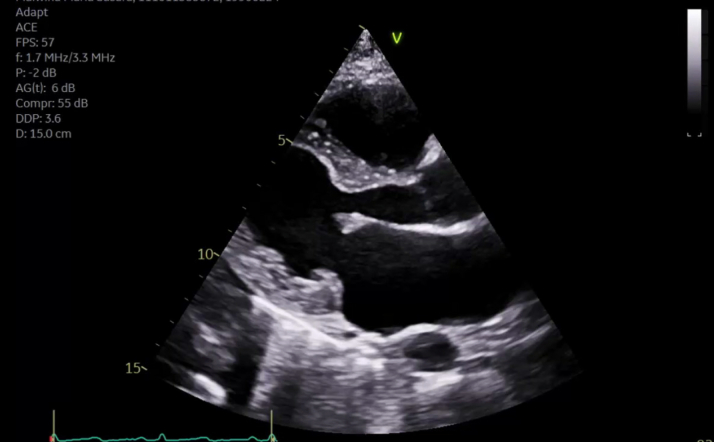
Transthoracic Echocardiography Parasternal Long Axis View Showing Resolution of Lesions

The patient was discharged with a plan for 6 weeks of intravenous antibiotic therapy and anticoagulation with warfarin. Follow-up TTE 3 weeks post-discharge showed complete resolution of the mobile echodensities in the LVOT, on the aortic valve, and in the RVOT (Figure 4). She was seen in clinic with no evidence of recurrence or residual symptoms from the stroke.

## Discussion

Intracardiac lesions in the context of blood culture–negative endocarditis (BCNE) pose significant diagnostic challenges, particularly when other possible causes of intracardiac lesions, such as thrombi, NBTE, and cardiac tumors, must be considered.^1^ In immunocompromised patients who are unable to mount a febrile response, the diagnostic complexity is further heightened.^1^ Although advances in imaging techniques have improved the characterization of intracardiac lesions, interpretation is often nuanced and requires careful clinical correlation.^2^

We describe a case of a patient with thrombophilia and on immunosuppression for lung transplantation who presented with a mobile echogenic mass in the LVOT attached to thickened interventricular septum and extending into the RVOT with aortic valve involvement. Approximately 90% to 95% of patients with IE have positive blood cultures, with 5% to 10% having BCNE often due to prior antibiotic use or the presence of fastidious organisms not detected by standard culture media.^1^^,^^3^ This case was atypical as the patient met only 1 major criterion of the modified Duke criteria (echocardiographic evidence) without any supporting minor criteria, making IE less likely.^4^ Echocardiography plays a crucial role in the diagnosis of IE, with the sensitivity of TTE and TEE for detecting vegetation at 75% and 90%, respectively.^5^ Occasionally, diagnostic uncertainty with TTE persists when clinical and imaging data are incongruent. In this patient, the modified Duke criteria for IE were not met, and neither TEE nor TTE revealed destructive valvular complications typically associated with IE. Thus, a plausible alternative diagnosis such as intracardiac thrombus or NBTE was strongly considered, especially given the history of thrombophilia and recurrent thrombotic events. NBTE was deemed less likely given the negative work-up for antiphospholipid syndrome, malignancy, and systemic lupus erythematosus;^6^ however, intracardiac thrombus remained a major consideration. In this case, the risk of prematurely discontinuing empirical antibiotic therapy outweighed the potential harm. Despite being on therapeutic anticoagulation, the patient subsequently developed a cardioembolic stroke and was found to have TTE findings of persistent lesions in the LVOT and a new lesion in the RVOT.

This case highlights the important role of PET/CT imaging in endocarditis, especially BCNE. Current guidelines recommend PET/CT with FDG as an adjunctive diagnostic tool in cases of suspected IE with unequivocal echocardiographic findings.^1^ Ultimately, higher morbidity and mortality are attributed to BCNE, often as a result of delayed diagnosis, delayed initiation of antibiotics, or premature discontinuation of antibiotics due to an alternative diagnosis, and this report underscores the critical need for a high index of suspicion and timely use of advanced imaging modalities in complex presentations.

## Conclusions

This case emphasizes the diagnostic complexity of intracardiac lesions in immunocompromised patients with underlying thrombophilia. Differentiating between IE and other IE masqueraders on echocardiography, such as NBTE, cardiac tumors, and intracardiac thrombus, can be confounded by inconclusive multimodal imaging and laboratory data. However, integrating clinical context with advanced imaging modalities such as PET/CT and maintaining a high index of suspicion for culture-negative endocarditis even in the absence of typical features remains critical.

## Funding Support and Author Disclosures

Dr Al-Kazaz has received research grant funding from Kiniksa Pharmaceuticals, Ventyx BioSciences, and Cardiol Therapeutics; speaking honoraria from Kiniksa Pharmaceuticals, and consulting fees from Edwards Lifesciences. All other authors have reported that they have no relationships relevant to the contents of this paper to disclose.
